# External Cerebrospinal Fluid Drainage in the Management of Nonaneurysmal Subarachnoid Hemorrhage

**DOI:** 10.7759/cureus.23423

**Published:** 2022-03-23

**Authors:** Ugo Ugwuanyi, Kenechukwu Igbokwe, Daniel E Onobun, Morayo Salawu, Chizimenu O Mordi

**Affiliations:** 1 Neurological Surgery, National Hospital Abuja, Abuja, NGA; 2 Neurological Surgery, Wellington Neurosurgery Centre, Abuja, NGA; 3 Neurology, Wellington Neurosurgery Centre, Abuja, NGA; 4 Anesthesiology, National Hospital Abuja, Abuja, NGA

**Keywords:** cerebrospinal fluid, external ventricular drainage, lumbar drainage, hydrocephalus, non-aneurysmal sah

## Abstract

Distinguishing the aneurysmal from nonaneurysmal subarachnoid hemorrhage (SAH) may be difficult as acute bleeding in the subarachnoid space is a common denominator. It is believed that toxic effects of breakdown products of acute bleed, including hemoglobin, contribute to the morbidity and mortality of this condition; and that early drainage will potentially reduce them. This series focuses on our local experience with the application of external cerebrospinal fluid (CSF) drainage in the management of a series of cases confirmed to be nonaneurysmal SAH and its effects on the outcome. The objective of this report is to observe the usefulness of external CSF drainage in the management of nonaneurysmal SAH. Five consecutive cases over four years were reviewed and reported as a case series. The main points we focused on were presentation, diagnostic findings on imaging, CSF drainage, and outcome up to six months. All the patients presented with headaches described as sudden, and only one had significant impairment of consciousness Glasgow Coma Scale (GCS) 10/15. Three out of the five patients had a premorbid hypertensive condition of unclear control status. We also observed that three out of the five had a low-pressure pretruncal/perimesencephalic pattern of bleed, whereas two had the typical high-pressure SAH pattern. CT angiography (CTA) was negative in all. Four had lumbar drainage, while one had external ventricular drainage. All were discharged within three weeks and functioned optimally at six months. CSF drainage in managing nonaneurysmal SAH is achievable with minimal access procedures, including lumbar drain (LD) and external ventricular drainage (EVD), which may have further reduced the low morbidity normally associated with this condition.

## Introduction

Clinical presentation of subarachnoid hemorrhage is often sudden, and the management is even more challenging, especially in a resource-poor setting. There is presently not a lot of local data and experience in the contemporary management of this inherently fatal form of stroke in our practice environment [1]. Nonaneurysmal type subarachnoid hemorrhage (SAH) has a lower rate of mortality and morbidity in comparison to the aneurysmal type. Irrespective of the type of SAH, they both have a challenge of an unusual amount of fresh blood in the subarachnoid space, which has been implicated in the emergence of complications such as cerebral vasospasm. This results in poor outcomes, especially in aneurysmal types and also, to a lower extent, in nonaneurysmal ones. Although the pathophysiological mechanisms of vasospasm are yet to be fully understood, we currently know that breakdown products of hemoglobin play a major role in this process [2]. It has also been observed that the incidence of vasospasm shows a close correlation with volume, density, and persistence of clots in the subarachnoid space and ventricular cavities [3]. In line with this idea, it has been suggested that early surgical removal of these clots may reduce the frequency and severity of vasospasms [4]. The bleed and its broken-down products are usually mixed with cerebrospinal fluid (CSF) at various concentrations depending on the volume of the bleed. Therefore, drainage of the CSF is a pragmatic way of cleansing the subarachnoid space of these bloodstains. This is also facilitated by the fact that fresh CSF is constantly produced and circulated at an average rate of 20ml/hour.

Several methods of CSF drainage have been reported, including external ventricular drainage, continuous lumbar drainage, and cisternal drainage with microsurgical fenestration of the lamina terminalis. The latter technique was recently reported but abandoned as it was shown to have no benefits [5]. An external ventricular drain (EVD) is useful in the drainage of CSF containing breakdown products of hemoglobin; however, this technique has been associated with an increased risk of developing ventriculitis and posthemorrhagic hydrocephalus [6]. Lumbar drainage (LD) is an alternative and has lower rates of hemorrhagic, obstructive, and infectious complications than ventricular drainage, and its use in patients with SAH is based on evidence provided by Macdonald [7] and Hänggi et al. [8]. These authors found that CSF drainage may reduce the incidence and potential consequences of vasospasms [7,8]. Some studies have revealed that due to gravitational forces, the concentration of blood components in CSF in patients with SAH is greater when CSF is collected using an LD than with an EVD. This proves that blood products accumulate mainly in the lumbar and basal cisterns [9]. These findings indicate that draining CSF from the lumbar cistern allows for more efficient clearance of breakdown products of hemoglobin from CSF and may therefore have detectable clinical benefits. 

Two distinct hemorrhage patterns have been described radiologically in the nonaneurysmal SAH: an aneurysmal hemorrhage pattern with no identifiable aneurysm on angiography and the pretruncal/perimesencephalic hemorrhage pattern [10]. Regardless of the cause of bleed, it is currently known that breakdown products of hemoglobin play a major role in the process of vasospasm which shows a close correlation with volume, density, and persistence of clots in the subarachnoid spaces and ventricular cavities. It is hoped that early deployment of external CSF diversion/drainage through external ventricular drainage or continuous lumbar drainage will encourage the early cleansing of the subarachnoid spaces, with an earlier resolution of symptoms in this category of patients and their subsequent return to normal activities. Following appropriate clearance from the institutional ethical review board and consent from patients, this report presents a concise review of a series of five patients, and the experiences gathered therefrom are hereby shared.

## Case presentation

Case 1

**Figure 1:**
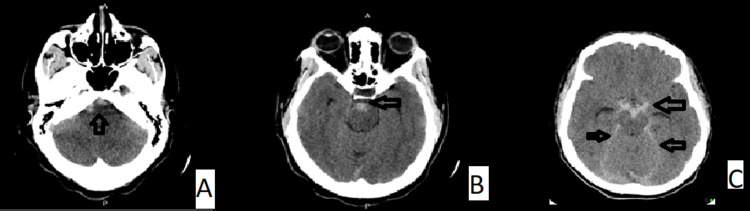
Axial slides of plain CT brain showing nonaneurysmal SAH into the prepontine, perimesencephalic, and interpeduncular cisterns.

**Figure 2:**
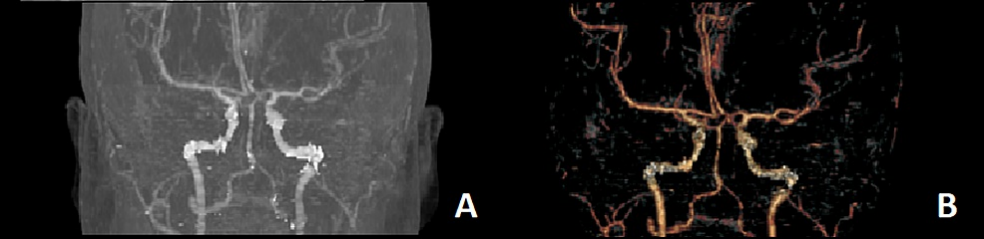
CT angiography showing no vascular abnormality in the posterior circulation.

A 34-year-old woman was referred from another facility on account of a one-week history of sudden onset of severe headache, blurring of vision, and episodes of projectile vomiting. There was no history of recent head trauma. She later described the onset of the headache as the worst headache of her life. She had been diagnosed with hypertension since her first pregnancy 10 years ago. In the ED, her blood pressure was 140/97mmHg. Pain visual analog score (VAS) was 9/10. An urgent CT of the brain demonstrated acute SAH in the prepontine and perimesencephalic cisterns, without extension into the sylvian or interhemispheric fissures. There was no intraventricular or intraparenchymal hemorrhage (Figure 1). CT angiography (CTA) did not reveal any aneurysms (Figure 2).

Her physical examination was notable for a right abducens palsy with nuchal tenderness. She was restless, in painful distress, but was conscious, obeyed commands, and was oriented to time, place, and person. She was unable to count fingers on the right eye but could perceive light movements. 

Laboratory examinations, including complete blood count, serum electrolytes, liver function tests, coagulation studies, and serologies, were all within normal limits.

Initial management was commenced with analgesics, nimodipine tablets 60mg four-hourly, liberal supply of intravenous fluids. On the second day after admission, she was still in severe painful distress, VAS score of 8-9/10. Under sterile conditions, continuous lumbar drainage was set at a pressure of 15cmH_2_O. Drainage was set up to drain at around 5-15cc per hour. She was on continuous lumbar drainage for 10 days. The patient improved clinically during the hospital stay and was discharged out of the ICU on post-bleed day 14, tolerating a full diet and ambulating independently. She was discharged home on the 16th post-bleed day. At the two-week follow-up after discharge, she had a head CT which demonstrated marked improvement in the perimesencephalic bleed. At the six-month follow-up, she was doing exceptionally well without a headache and had a completely normal examination. She returned to her work and all premorbid daily living activities.

Case 2

The second patient, a 54-year-old woman, was referred to our facility with complaints of a sudden onset of severe headaches and neck pain that had begun 12 hours before. Her symptoms were associated with an episode of projectile vomiting and dizziness. She was not known to be hypertensive. On examination, she was fully conscious oriented in time, place, and person but was in painful distress with an admitting blood pressure of 160/70mmHg and a positive Kernig’s sign. She had no focal neurologic deficits.

An urgent brain CT showed an acute subarachnoid bleed in the perimesencephalic region, but no aneurysm was identified on a follow-up CT angiogram. Laboratory examinations, including complete blood count, serum electrolytes, liver function tests, coagulation studies, and serologies, were all within normal limits.

Initial management included admission to the intensive care unit (ICU) and commencement of analgesics, sedation with midazolam-fentanyl in a syringe pump, nimodipine tablets 60mg four-hourly, and intravenous fluids at maintenance of 3L/day. On the second day after admission, she was still in severe painful distress, pain VAS score of 9-10/10, which informed the decision to establish continuous lumbar drainage, which was discontinued after five days following satisfactory clinical improvement and clearance of blood-stained CSF drainage. She was discharged from the ICU on post-bleed day seven, tolerating a full diet and ambulating independently. She was discharged home on the 14th post-bleed day with residual symptoms of mild headaches, VAS of 3/10. After discharge, she had a head CT at the third-month follow-up, which demonstrated marked improvement in the perimesencephalic bleed. Her most recent magnetic resonance angiogram (MRA) showed no features of an aneurysm. At the six-month follow-up, the modified Rankin Scale (MRS) was 0/6, and the patient was functioning independently with no disability.

Case 3

A 52-year-old woman was referred to our facility 10 hours after the onset of symptoms, with complaints of a sudden onset of severe headaches and an episode of loss of consciousness, which lasted about two minutes. She is not known hypertensive. On examination, she was fully conscious oriented in time, place, and person but was in painful distress with an admitting blood pressure of 175/80mmHg. She had no focal neurologic deficits.

An urgent brain CT showed an acute subarachnoid bleed in the perimesencephalic region similar to Figure 1, but no aneurysm was identified on a follow-up CT angiogram. Laboratory examinations, including complete blood count, serum electrolytes, liver function tests, coagulation studies, and serologies, were all within normal limits.

Initial management included admission to the ICU and commencement of analgesics, nimodipine tablets 60mg four-hourly, and intravenous fluids at maintenance of 3L/day. Under sterile conditions, continuous lumbar drainage was set at a pressure of 15cmH_2_O. Drainage was set up to drain at around 5-15cc per hour. She was conservatively managed in the ICU, and clinical improvements were noted. She was discharged from the ICU on post bleed day six after removing the lumbar drainage. She was tolerating a full diet and ambulating independently. The daily output of the lumbar drainage was between 246-280ml. She was discharged home on the 14th post-bleed day with very mild residual headaches, VAS of 3/10. At the first follow-up visit two weeks after discharge, CTA showed complete disappearance of previously noted perimesencephalic bleed and no identifiable aneurysm. At the six-month follow-up, MRS was 0/6, and the patient was functioning independently with no disability.

Case 4

**Figure 3:**
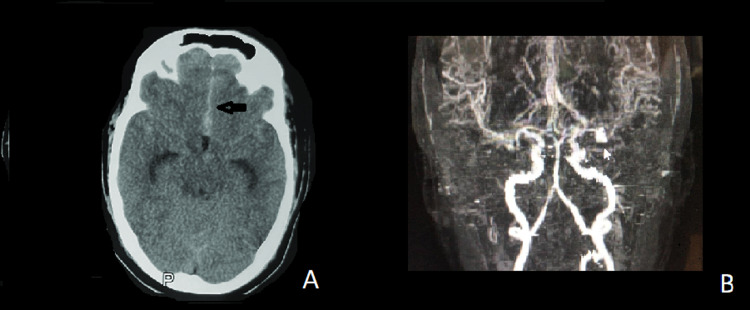
(A) Axial slide of plain CT brain showing nonaneurysmal SAH into the anterior interhemispheric fissure and dilated both temporal horns of lateral ventricles. (B) CT angiography showing no vascular abnormality in the anterior circulation.

A 45-year-old man was referred to our facility with complaints of a sudden onset of severe headaches, altered consciousness, and vomiting of a day’s duration. He was a known hypertensive not adherent to his prescribed medications. On examination, he was restless, Glasgow Coma Scale (GCS) 10/15 (E4, V1, M5), with an admitting blood pressure of 195/100mmHg. Pulse rate was 62bpm, and he saturated at 98% on room air. An urgent CT of the brain (Figure 3A) showed an acute subarachnoid bleed in the anterior interhemispheric fissure and sylvian fissures bilaterally and early hydrocephalus. Despite this typical aneurysmal SAH pattern of bleed, CT angiogram (Figure 3B) did not reveal a clear aneurysmal source of the subarachnoid bleed. A clinical diagnosis of World Federation of Neurological Societies (WFNS) grade 4 with no clear source of aneurysm was made. Initial management was neurocritical, involving sedation, intubation, oxygen support, adequate pain control, and continuous multimodality monitoring. In view of the uncontrollably rising intracanial pressure (ICP) consequent upon the impaired CSF flow due to blood clots in the ventricular and subarachnoid space, an external drainage of CSF was carried out via a lumbar-drain insertion. Continuous lumbar drain output was monitored hourly until the initially serosanguinous drainage became crystal clear on the 7th post-bleed day, then it was discontinued and removed. Blood pressure control was achieved with atenolol, amlodipine, and valsartan. His cognitive and higher mental functions improved satisfactorily. A repeat CTA and MRA still did not reveal any convincing evidence of an aneurysm, and the patient was discharged to outpatient follow-up 13 days after admission. At the six-month follow-up, MRS was 0/6 and he was functioning independently with no disability.

Case 5

**Figure 4:**
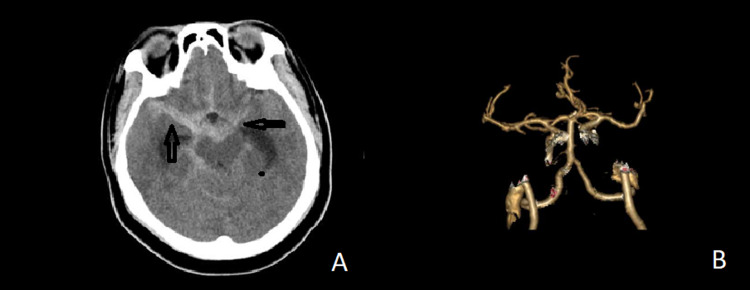
(A) Axial slide showing subarachnoid bleed with intra-ventricular extension and acute obstructive hydrocephalus. (B) CT angiography showing no vascular abnormality in the posterior circulation.

A 34-year-old female was referred to our facility with complaints of headache, vomiting, and neck pain of 12 hours duration. She was a known hypertensive, poorly compliant on anti-hypertensive medications. On examination, she was agitated and restless. GCS of 15/15 with admitting blood pressure of 210/110 mmHg, pulse rate of 70bpm, and saturation of 98% in room air. An urgent CT (Figure 4A) scan showed subarachnoid bleed with intraventricular hemorrhage (IVH) extension and acute obstructive hydrocephalus. Again, despite this typical aneurysmal pattern of SAH bleed, CT angiography (Figure 4B) did not reveal any clearly defined aneurysm although a posterior communicating artery (PCOM) aneurysm was suspected. A diagnosis of SAH, IVH, and acute obstructive hydrocephalus (AOHCP) was made. Initial management involved neurocritical care monitoring. She had an external ventricular drain inserted on the day of presentation; however, it needed to be revised 11 days after. A repeat CTA at this time was unequivocal on the absence of any aneurysmal source of the initial bleed. However, the ventricles displayed persisting communicating hydrocephalus and was later converted to a ventriculoperitoneal (VP) shunt due to failure to wean off EVD. She subsequently did well and was discharged home on the 19th day on amlodipine, valsartan, hydrochlorothiazide, and labetalol. Repeat CTA at one-month follow-up did not reveal any aneurysm. At the six-month follow-up, MRS was 0/6, and the patient was functioning independently with no disability.

## Discussion

Aneurysmal SAH is more common than nonaneurysmal SAH. No aneurysm is detected in 15-20% of SAH on the first angiogram [11]. This percentage is consistent with the knowledge that nonaneurysmal type comprises up to 15% of all SAH, as seen in studies by Ugwuanyi et al. [1], Maslehaty et al., [12], Ildan et al. [13], and Gupta et al. [14].

Two patterns of blood deposition in the subarachnoid space in nonaneurysmal bleeds as reflected on neuroimaging (CT scan) have been long recognized as follows: above the Liliequist’s membrane (LM) and concentrated on the chiasmatic, interhemispheric, sylvian fissures, carotid cisterns, etc. and below the LM and concentrated on the crural, interpeduncular, and prepontine cisterns. Anatomically, the Liliequist’s membrane separates the prepontine and interpeduncular cisterns from the chiasmatic cisterns [15]. The LM is further characterized by a thicker and more resilient superior leaflet (diencephalic membrane) which separates the chiasmatic cistern above from the interpeduncular and prepontine cistern below, whereas the flimsier inferior membrane separates the interpeduncular from the prepontine cisterns [15]. Consequently, low-pressure bleeds, which are usually from mesencephalic veins, tend to accumulate below the inferior layer of LM (mesencephalic membrane), extending to the interpeduncular, crura, and the carotid cisterns. Conversely, high-pressure bleeds have a predilection to the chiasmatic cisterns and above and are contained by the tougher diencephalic LM [16].

Irrespective of the bleeding source, the common denominator is the presence of acute blood in the subarachnoid compartment which should normally contain pure CSF bathing the blood vessels and pia matter. The result is exposure to known sequelae and complications of such an anomaly, including vasospasm with delayed ischemic neurological deficits, hydrocephalus, electrolyte derangements, rebleeding, etc. Early drainage of this blood and hemoglobin contaminated space has been documented by Macdonald [7]and Hänggi et al. [8] to reduce the incidence and severity of these complications and improve outcomes.

Table 1 summarizes the five cases presented here. This condition was more common in females (4:1) with an average age incidence of 43.8yrs. All the patients presented with headaches described as sudden and worse in a long time, but only one had significant impairment of consciousness (GCS 10/15). Three out of the five patients had premorbid hypertensive condition of unclear control status. We also observed that three out of the five had a pretruncal/perimesencephalic pattern of bleed, whereas two had the typical high-pressure SAH pattern of bleed on the basal, interhemispheric, and chiasmatic cisterns clearly above the diencephalic portion of the Lilliquist’s membrane. But what was common to all bleed patterns was the absence of any demonstrable aneurysms on both initial and subsequent angiogram studies. External CSF diversion was performed on all cases promptly, and four out of the five had continuous lumbar drainage. In contrast, only one had initial external ventricular drainage, which was subsequently converted to a permanent VP shunt. The predominant choice of the continuous lumbar drainage was predicated on the earlier reports, which revealed that due to gravitational forces, the concentration of blood components in the lumbar subarachnoid spaces allows for more efficient clearance and may therefore have detectable clinical benefits [9]. 

**Table 1:** Summary of five patients treated for nonaneurysmal SAH at Wellington Neurosurgery Centre over four years HA: headache, HTN: hypertension, SAH: subarachnoid hemorrhage, IVH: intraventricular hemorrhage, HCP: hydrocephalus, CSF: cerebrospinal fluid, CLD: continuous lumbar drainage, EVD: external ventricular drainage, VP: ventriculoperitoneal, LOC: loss of consciousness, GCS: Glasgow Coma Scale, MRS: modified Rankin Scale, M: male, F: female, NCCT: non-contrasted computer tomography scan, CTA: CT angiography, NAD: no abnormality detected, NA: not applicable

SN	Age	Sex	Presentation and Duration	NCCT	CTA	External CSF Diversion and Duration	VP Shunt	Hospital Stay (days)	Six-month Outcome (MRS)
1	34	F	HA, poor vision, neck pain, vomiting, HTN, GCS: 15; Duration: 7 days	Prepontine, Perimesencephalic	NAD	CLD: 10 days	NA	16	0/6
2	54	F	HA, neck pains, GCS: 15; Duration: 12 hrs	Perimesencephalic	NAD	CLD: 5 days	NA	14	0/6
3	52	F	HA, LOC (transient), GCS: 15 (on admission); Duration: 10 hrs	Perimesencephalic	NAD	CLD: 6 days	NA	14	0/6
4	45	M	HA, LOC, vomiting, HTN; Duration: 24 hrs	Typical SAH, interhemispheric bleed, sylvian fissures bleed, IVH, HCP	NAD	CLD: 7 days	NA	13	0/6
5	34	F	HA, vomiting, neck pains, HTN; Duration: 12 hrs	Typical SAH, blood in basal cisterns, IVH, HCP	NAD	EVD: 11 days	Yes	19	0/6

It is interesting that most patients did not need the lumbar drainage beyond 10 days as evidenced by near-complete resolution of predominant symptoms of headache from admitting (VAS of 9-10/10 to 1-2/10) and restoration of the CSF color to crystal clear. The choice of external ventricular drainage option in one case was based on the large volume of blood seen on the four ventricles. It was therefore not surprising that this case did not only require re-siting the EVD on the contralateral side after 10 days in order to complete the drainage process to achieve clear CSF but also to discourage ventriculitis; he also required permanent CSF diversion with VP shunt insertion due to failure to wean off EVD and persistent communicating hydrocephalus.

Inspite of repeat angiograms at discharge and subsequent outpatient visits up to six months, no demonstrable aneurysm was spotted in any of the cases. There are several reasons why an aneurysm may fail to show on angiogram, including but not limited to degradation and poor-quality images, thrombosis and obliteration of aneurysm after hemorrhage, and too small an aneurysm to be visualized [17]. There are also bleed sources that fail to show on angiograms, such as cryptic vascular malformations, venous angiomas, cavernomas, and pretruncal SAH [16].

One patient had the longest hospital stay of 19 days, and the rest four left the hospital within two weeks of admission. However, the main presenting symptoms of headaches, neck pains and restlessness disappeared much earlier (10 days and five days, respectively). The reason for overall longer hospital stays were due to non-clinical factors. Overall clinical and neurological improvement were sustained even at six-months' follow-up with all returning to their work and previous activities of daily living. This is a further attestation to the already established low mortality and morbidity attached to these nonaneurysmal bleeds [18].

## Conclusions

CSF drainage in the management of nonaneurysmal SAH is achievable with minimal access procedures as outlined above. We know the already established fact that nonaneurysmal SAH comparatively runs a less stressful course. Therefore, the intent of this article is not to change established practice standards. As a matter of fact, it lacks the numbers to drive the statistical power to address any change in existing standards. Instead, we have presented our local experience that interventions directed at draining CSF contaminated with acute bleed and its broken-down products could potentially hasten resolution of distressing symptoms such as headache, neck pains, etc. for earlier improvement of comfort of these patients, thus facilitating early return to independent activities of daily living.
